# Academic publisher guidelines on AI usage: A ChatGPT supported thematic analysis

**DOI:** 10.12688/f1000research.142411.2

**Published:** 2024-01-16

**Authors:** Mike Perkins, Jasper Roe

**Affiliations:** 1 British University Vietnam, Hanoi, Vietnam; 2 James Cook University Singapore, Singapore, Singapore

**Keywords:** Generative AI, Academic Integrity, Inductive Thematic Analysis, Publisher Policies, Research Methodologies, AI-Assisted Authorship, Ethical Guidelines, Academic Publishing

## Abstract

**Background:**

As Artificial Intelligence (AI) technologies such as Generative AI (GenAI) have become more common in academic settings, it is necessary to examine how these tools interact with issues of authorship, academic integrity, and research methodologies. The current landscape lacks cohesive policies and guidelines for regulating AI’s role in academic research which has prompted discussions among publishers, authors, and institutions.

**Methods:**

This study employs inductive thematic analysis to explore publisher policies regarding AI-assisted authorship and academic work. Our methods involved a two-fold analysis using both AI-assisted and traditional unassisted techniques to examine the available policies from leading academic publishers and other publishing or academic entities. The framework was designed to offer multiple perspectives, harnessing the strengths of AI for pattern recognition while leveraging human expertise for nuanced interpretation. The results of these two analyses are combined to form the final themes.

**Results:**

Our findings indicate six overall themes, three of which were independently identified in both the AI-assisted and unassisted, manual analysis using common software tools. A broad consensus appears among publishers that human authorship remains paramount and that the use of GenAI tools is permissible but must be disclosed. However, GenAI tools are increasingly acknowledged for their supportive roles, including text generation and data analysis. The study also discusses the inherent limitations and biases of AI-assisted analysis, necessitating rigorous scrutiny by authors, reviewers, and editors.

**Conclusions:**

There is a growing recognition of AI’s role as a valuable auxiliary tool in academic research, but one that comes with caveats pertaining to integrity, accountability, and interpretive limitations. This study used a novel analysis supported by GenAI tools to identify themes emerging in the policy landscape, underscoring the need for an informed, flexible approach to policy formulation that can adapt to the rapidly evolving landscape of AI technologies.

## Introduction

The emergence of Generative Artificial Intelligence (GenAI) tools, exemplified by OpenAI’s ChatGPT, has resulted in a significant shift in the academic research landscape. These tools, powered by Large Language Models (LLMs) such as GPT3.5 and GPT-4, are capable of generating human-like text with impressive fidelity, accuracy, and creativity ( Cotton *et al.*, 2023; Perkins, 2023a). The advent of these tools has necessitated a closer examination of the academic integrity issues regarding how they are used by researchers. As GenAI has become more sophisticated and widely adopted in academia, understanding its implications for research integrity and establishing appropriate usage policies and guidelines has become increasingly important. Although there are multiple perspectives on what constitutes research integrity ( Shaw & Satalkar, 2018), in this study, we consider research integrity to refer to the trustworthiness of the research process itself, from conceptualisation to publication. It is also crucial to understand how these tools support the academic research process and how to develop effective strategies and policies to ensure the ethical use of these technologies.

The advent of digital technology has profoundly impacted academic integrity. Over the past decades, instances of academic misconduct, notably plagiarism, have declined ( Curtis, 2022). This positive trend is largely attributable to the role of technological and educational measures in mitigating plagiarism. However, the rise of advanced AI tools, notably Automated Paraphrasing Tools (APTs) and GenAI tools, has introduced new complexities. These tools, which are capable of generating and rephrasing content, facilitate subtle forms of plagiarism that challenge traditional detection methodologies ( Perkins, 2023a). This has further escalated a technological ‘arms-race’ ( Cole & Kiss, 2000; Mortati & Carmel, 2021; Roe & Perkins, 2022; Eaton, 2022) in plagiarism detection, with new tools and techniques being developed to keep pace with the evolving capabilities of AI.

The rapid advancement of AI technologies and their increasing application in various domains has underscored the need for comprehensive policy analysis and ethical considerations. Despite the widespread use of digital and AI technologies, only a small percentage of higher education institutions (HEIs) have developed formal policies surrounding their use since the launch of ChatGPT ( Perkins & Roe, 2023; Xiao *et al.*, 2023). Just as HEIs had to quickly develop policies and guidelines on students’ use of GenAI tools, academic publishers were also forced to consider how to manage the use of GenAI tools by authors. The uncertainty still present in the academic landscape in this area highlights the pressing need for additional research on how academic institutions, publishers, and employers must consider how these tools and technologies are being used by academics. The development and implementation of effective policies can guide the ethical use of AI tools more broadly in academic studies and research, ensuring that they are used to uphold academic integrity. Furthermore, a clear understanding of these policies can help researchers navigate the complex landscape of AI-assisted research, enabling them to leverage the benefits of these tools while avoiding potential pitfalls.

### Objectives and structure of the study

In this study, we review the policies of 107 publishers who are key stakeholders in the production of scholarly work to identify views regarding the use of GenAI tools. Using inductive thematic analysis, we carry out a detailed exploration of the 28 policies which mention GenAI tools. We dissect these policies to identify key themes across different publishers and discern commonalities and variances, thereby enabling us to better understand the collective stance on the use of GenAI tools in academic research. The insights gained from this policy analysis will not only highlight existing norms, but will provide actionable data that can guide the development of more unified and effective policies for the responsible use of GenAI in academia.

To assess the potential role of GenAI tools in academic research, this study adopts a dual-method analytical approach, employing both GenAI-assisted, and manual thematic analyses. Manual analysis refers to a ‘traditional’ approach to qualitative research, using a standard software package (QDA Miner) to assist with data coding and interpretation, with no use of GenAI tools. The aim is to provide empirical evidence to evaluate the effectiveness of GenAI tools, such as ChatGPT, as a research assistant or co-pilot within the academic research landscape. These analyses are designed to determine whether insights generated by GenAI are not only comparable, but may also exceed those obtained through human-led analysis, thereby providing evidence-based insights into the capabilities of GenAI tools for areas of academic research that have not previously been explored. Therefore, this study contributes to the ongoing discourse on the use of GenAI tools in academic research, especially as this particular use of GenAI tools in the research process is in direct contrast to the policies of several academic publishing entities, including the Social Sciences Research Network ^1^ (SSRN) and Edward Elgar ^2^.

The key objectives of this study are as follows:

1. To evaluate the role, capabilities, and effectiveness of GenAI tools such as ChatGPT in academic research by comparing the quality of insights generated through GenAI-assisted and manual thematic analyses.
2. To provide empirical evidence on how GenAI can be responsibly and effectively integrated into the academic research process.
3. To identify emergent themes, commonalities, and variances in academic publishing entities’ stances on the use of GenAI tools in scholarly works.
4. To generate actionable insights that will guide the development of effective and unified policies for the responsible use of GenAI tools in academic research, thereby contributing to the broader discourse on maintaining academic integrity in the era of advanced AI.

## Literature review

### The evolution and impact of artificial intelligence

Artificial Intelligence (AI) relates to the scientific field focused on developing intelligent machines which can reason, communicate, learn, plan, and solve real-world problems for the benefit of society ( Debrah *et al.*, 2022). Although AI has been an established field of scientific enquiry for the past sixty years, towards the end of 2019, it began to receive a slew of attention in popular culture. This is attributable to the rapid advancement of computing and processing power, which is currently outstripping Moore’s Law and doubling capacity every six months ( Pichai, 2023). The pace of change is so great that major corporations involved in developing AI have warned of the potential of a ‘superintelligence’ being developed within the decade ( OpenAI, 2023a). AI has a potentially transformative effect on the global level, leading to changes in world politics, military power, and warfare ( Johnson, 2019). At the individual level, it has been posited that the near future will see the merging of human biology and AI technologies, leading to a symbiotic relationship ( Jiang *et al.*, 2022).

### Ethical and regulatory considerations in AI development and use

The release of Generative AI (GenAI) tools and chatbots, such as OpenAI’s ChatGPT and Google’s Bard, has further stoked conversations around the risks and benefits of AI in multiple contexts. Noteworthy examples include thought leaders such as Google’s CEO Sundar Pichai warning us to ‘brace for impact’ ( Pichai, 2023, as cited in Elias, 2023), and the Centre for AI Safety releasing a statement signed by scientists and subject matter experts asking for a halt on AI development and identifying that the risk of human extinction from AI should be seen as a top global priority ( CAIS, 2023). Other, less critical, yet important risks include the security and protection of confidential data and the potential of training data containing biases or discriminatory ideologies ( King, 2023). As a result, governments and intergovernmental organisations worldwide are taking action on AI. The European Union is currently in the process of passing the AI Act, which represents the first comprehensive set of legislation regulating AI ( European Parliament, 2023). This framework prohibits high-risk applications of AI while providing guidelines for language models, such as ChatGPT. It mandates the disclosure of AI-generated content, prohibits the production of illegal content, and requires the publication of summaries for copyrighted data used in training ( European Parliament, 2023).

### The economic and sectoral impact of AI

Despite the serious risks outlined above and the push to build and implement regulatory frameworks, AI continues to pick up speed in the private sector. The market value of AI is anticipated to reach $190 billion USD by 2025, and it is having a revolutionary effect on multiple industries, including manufacturing, healthcare, education, transport, and medicine ( Jiang *et al.*, 2022). In factory and industrial production, AI enables self-monitoring, autonomous operations, and improved efficiency, with reduced downtime ( I. Ahmed *et al.*, 2022). In the field of healthcare, AI plays a major role in advancing nanomedicine for cancer treatment ( Tan *et al.*, 2023) and shows the potential for taking over traditionally human-performed tasks, such as the generation of medical discharge reports ( Patel & Lam, 2023). AI tools have also played a role in identifying case clusters and predicting infection models in the COVID-19 pandemic ( Peng *et al.*, 2022), and AI techniques are also being employed for drug discovery, identification of risk factors affecting patients, and disease diagnosis ( Kumar *et al.*, 2023). In architecture, AI contributes to sustainability in green construction practices ( Debrah *et al.*, 2022), and the finance sector is beginning to utilise AI in areas such as anti-money laundering and behavioural finance ( S. Ahmed *et al.*, 2022), while in relation to farming and food production, AI is being explored for its potential to address animal welfare issues ( Bao & Xie, 2022). In the newspaper publishing industry the German tabloid Bild has even reduced its workforce with the goal of replacing some job roles with AI ( Henley, 2023).

### AI’s role and ethical implications in academic research

Academia is also coming face-to-face with the disruptive potential of AI. AI-generated submissions to academic journals have been described as a ‘coming tsunami’ ( Tate *et al.*, 2023) and it has been claimed that publishers need to anticipate the potential of wholly AI-generated submissions ( Anderson *et al.*, 2023). To date, much of the focus in academia has been on the use of GenAI tools such as ChatGPT, thanks to their human-like text production capabilities ( Perkins, 2023a; Perkins & Roe, 2023), although other AI tools such as Elicit have also shown promise in summarising literature and identifying source material ( Roe *et al.*, 2023). Such capabilities have fuelled the debate on the positive and negative impacts of GenAI on scholarly work. Some researchers have reportedly co-authored studies with ChatGPT ( Pavlik, 2023) and produced papers in which the majority of the text was AI-generated (cf: Biswas, 2023; Cotton *et al.*, 2023). LLMs can be used not only for writing but also for summarising literature, generating titles, locating data sources, and identifying gaps in research ( Hutson, 2022; Nguyen-Trung *et al.*, 2023). In the classroom context, LLMs may increase the efficiency of assessment creation and provide personalised feedback for learners ( Baidoo-Anu & Owusu Ansah, 2023). On the side of publishers, AI tools may be able to assist in identifying suitable reviewers for academic work ( Solomon *et al.*, 2023), and concerns have been raised about the lack of editorial policies in using GenAI tools in the peer review process ( Garcia, 2023).

These AI models also carry implications for academic integrity ( Perkins, 2023a; Roe et al., 2023). At present, LLMs cannot completely compose a novel academic text or author a research study in the way a human researcher can because although they excel at human-like text production and organisation, they still lack the ability to generate novel ideas or engage in creativity ( Hammad, 2023). Furthermore, at this stage, using a GenAI tool like ChatGPT to draft a scientific paper still requires expert supervision, as text generated by AI is claimed to lack the nuances of human-authored text ( Salvagno *et al.*, 2023). However, LLMs offer students the opportunity to misrepresent their authorship and engage in surface-level learning when completing written assessments ( Cotton *et al.*, 2023; Perkins, 2023a). Likewise, non-academic authors may also pass off GenAI texts as their own work. As a result, efforts have been made to detect AI-generated text, although present studies show that humans are not adept at identifying AI-generated texts. In one study, reviewers of academic abstracts were only able to identify 68% of the content generated by ChatGPT, with a 14% false-positive rate ( Gao *et al.*, 2022). Even advanced AI detection software tools struggle, with a detection rate of only 54.8% in examples of work submitted to the Turnitin platform ( Perkins *et al.*, 2023), and inaccuracies in a range of detection tools have been identified and discussed by Weber-Wulff *et al.* (2023). As a result, such cases have spurred the need for author guidelines on AI use in scholarly publishing ( Liebrenz *et al.*, 2023). As LLMs continue to develop, it has been envisioned that their capabilities will extend to other tasks, such as generating data visualisations and summaries of quantitative and qualitative data with minimal user input, potentially revolutionising aspects of the research process. This has been demonstrated with the beta release of OpenAIs ‘Advanced Data Analysis’ mode of ChatGPT which has the ability to use built in Python libraries to extend the abilities of the GPT4 model beyond language-based tasks ( OpenAI, 2023b).

At the same time, authors need to be made aware of the potential harm to their work when using LLMs. It is possible that their use could perpetuate prejudice and bias found in their training data ( Lund *et al.*, 2023). Researchers should also consider that there is a risk of escalating inequality; the efficiency-enhancing effects of LLMs could contribute to the growth of the ‘Matthew Effect’, in which successful researchers receive more attention and citations, while newer scholars struggle to receive attention for their work ( Lund *et al.*, 2023). On the other hand, the use of such tools could contribute to a more level-playing field. Some believe that the evolution of the scholar’s role could pivot towards asking the right questions rather than only articulating conclusions ( Jabotinsky & Sarel, 2022). In this scenario, if the demanding processes of writing, reviewing literature, and presenting results are taken care of by an LLM or AI tool, then perhaps there is more scope for scholars to focus on other aspects of research quality.

At the core of the issue of research integrity relating to AI and LLM use is authorship and whether non-human actors can claim authorial rights and obligations in the production of text. In Pavlik’s (2023) case, ChatGPT was listed as the second author, and this symbolises some of the areas of debate and confusion. The discussion around AI-generated text and authorship is not new but dates to the production of an article by a program named Racter in 1981 ( Lee, 2023). Lee (2023) highlights that from a legal perspective, there are varying stances on non-human authors’ credibility. In China, for example, copyright law does not necessitate that an author is a person, while in the USA and Korea, it does. Examples of this issue playing out in popular media have included the rights of a self-taken photograph by a macaque, a copyright claim which was eventually rejected in the USA ( Lee, 2023). While a useful and interesting point of reference, copyright law is distinct from academic ethics, and it seems that at the time of writing the consensus among academic publishers is that tools such as chatbots do not meet authorship criteria, as they cannot be held accountable for the produced text ( Lee, 2023). However, the issue has not been resolved, and there have been calls for scholarly organisations such as the APA to draft clear guidelines on the use of AI-generated text in research ( Tate *et al.*, 2023).

The reasons why LLMs are not able to hold authorship roles are best exemplified by the criteria given by the Committee on Publication Ethics (COPE). COPE states that four criteria must be met to attribute authorship: substantial contribution, critical revision, final approval, and agreement on accountability of the work ( Pourhoseingholi *et al.*, 2023). LLMs can contribute substantially and engage in the revision of work, but they cannot hold responsibility or accountability for the work. However, this does not mean that LLMs and AI tools should be banned. Rather, ethical guidelines must be in place to govern their use ( Rahimi & Talebi Bezmin Abadi, 2023). To this point, some academic journals are encouraging the use of AI to refine manuscripts and edit papers, provided that the technology’s contribution is appropriately recognised ( Crawford *et al.*, 2023). However, these guidelines can only go so far, given that accurately identifying text produced by tools such as ChatGPT remains a major challenge ( Hu, 2023; Weber-Wulff *et al.*, 2023; Perkins, Roe *et al.*, 2023; Anderson *et al.*, 2023).

This study makes two unique contributions to the existing literature. First, it undertakes a comparative analysis of publisher policies on the usage of Artificial Intelligence, an area that has hitherto not been formally explored. Such an examination is critical for understanding the formal stances taken by academic journals, publishers, and other entities involved in research ethics, thereby offering valuable insights into the evolving norms and guidelines that shape AI’s role of AI in scholarly work. Second, the study itself employs GenAI tools in both data analysis and writing up, thus serving as a practical demonstration of GenAI’s capabilities as a ‘co-pilot’ in academic research. This dual focus not only enriches our understanding of the academic publishing landscape in the context of AI, but also validates the utility of GenAI tools in facilitating rigorous academic enquiry.

## Methods

The primary analytical tool used in this study is inductive thematic analysis, a method widely used in qualitative research for identifying, analysing, and reporting patterns (themes) within data ( Braun & Clarke, 2006). Thematic analysis offers a flexible and useful research tool that can potentially provide a rich and detailed yet complex account of data ( Braun & Clarke, 2006). In this study, thematic analysis is used to classify policies based on emergent themes. Thematic analysis in policy research is a well-established method that provides a systematic approach for analysing qualitative data ( Nowell *et al.*, 2017). It involves the identification of themes or patterns of meaning that arise repeatedly within the data. These themes are then used to draw conclusions about the phenomena being studied. In this study, the phenomena in question are the policies of academic publishing houses regarding the use of AI in academic research.

To identify policies to be analysed, existing lists and rankings of academic publishers found online ( Nishikawa-Pacher, 2022; Wikipedia, 2023) were combined to form a master list of academic publishers which would be investigated. These publishers included traditional scholarly publishers, preprint servers, university publishing houses, general book publishers, newspapers, academic integrity focus groups, and NGOs. The master list was edited to remove known or suspected instances of publishers of predatory journals as identified by the most recently available version of Beall’s List, most recently updated in 2021 ( Beallslist.net, 2021). This list was then supplemented by web searches including terms such as ‘ *AI/ChatGPT journal/publisher policies*’ to identify any journals, publishers, or publishing related groups/institutions which were found to have some policy related to the use of GenAI tools. This resulted in a master list of 107 entities for review.

Manual searches were then conducted on the websites of all entities to explore whether they had a policy in place relating to the use of Generative AI tools. From this manual search, 36 entities were identified as having an AI policy, eight of these were replicas. Replicas were due to either the use of wording from other entities, such as The Committee of Publication Ethics (COPE), or because the publishing houses were imprints of broader publishing groups, and therefore did not have their own policy. This resulted in a final list of 28 unique policies for analysis.

A thematic analysis involves several steps. First, the data is familiarised through reading and re-reading. Next, initial codes are generated, which are then searched for themes. These themes are then reviewed and defined. Finally, the report is produced, which in this case is an analysis of the policies regarding the use of AI in academic research. In the context of this study, thematic analysis is particularly useful for classifying policies based on emergent themes. This approach allows for the identification of commonalities and differences in the policies of different institutions and publishers, providing a comprehensive overview of the current state of policy regarding the use of AI in academic research. The use of thematic analysis in this study is guided by the principles outlined by Braun and Clarke (2006). This involves a six-phase process of familiarisation with the data, generating initial codes, searching for themes, reviewing themes, defining and naming themes, and producing a report. This rigorous approach ensures a thorough and systematic analysis of policies, providing valuable insights into the current state of policy regarding the use of AI in academic research.

In the analysis, we combined coding, analysis, and interpretation with an analysis conducted with assistance from the GenAI tool ChatGPT Plus (using the underlying LLM of GPT-4). In our methodological process, these analyses were conducted separately by the two researchers, and the results were then compared during the final stages of the research process, from reviewing themes to producing the report. Thematic analysis without the support of AI software was conducted by Researcher 2 (R2), and Researcher 1 (R1) conducted the analysis with GenAI assistance. The rationale for this approach was first to develop greater insight into the data and determine whether there were significant differences between LLM-supported and traditional data analysis and interpretation processes, and second, to engage in the use of AI in academic research. In this sense, the research demonstrates a way of using AI to support and assist, yet not author, academic work. After combining the results of the two analyses, the final set of themes was selected.

## Results

In the following, we begin by presenting the results obtained through traditional, manually generated thematic analysis. This is then contrasted with the initial results obtained through the GenAI tool analysis, before our final set of themes is described. All quotes used in the below results are drawn verbatim from academic publisher policies available online. To preserve the record of the policy wording when they were originally downloaded, the publisher policies and URLs can be found in the associated data for this paper ( Perkins, 2023b). The full policy statement for each publisher/journal is shown in column D, with the URL shown in Column H.

### Results section one: Manually generated thematic analysis

The thematic analysis with no AI assistive tools was developed using the QDA Miner software by R2. R2 began by reading through the policies in detail, searching for indicators of interest, and identifying features that typified the ‘qualitative richness of the phenomenon’ ( Boyatzis, 1998, p.1) prior to iteratively and reflexively refining them. Coding took place at the semantic level, looking at surface features of the text rather than trying to identify deeper meanings at the latent level. This choice was made based on the genre of the text: policy guidelines are designed to be explicit, rather than implicit. The coding process was performed in a traditional manner, using a standard software package for qualitative analysis (QDA Miner). Initial steps involved allocating codes to specific passages. For example, ‘Authors must take full responsibility’ was a relevant recurring code throughout the policies, and this code was later translated into the broader theme of ‘authorship is human’. Although it may seem that publisher policy documents would be formulaic and straightforward, with little room for deeper analysis, three key themes were identified from the data. The themes identified from the manual analysis are visible in Table 4, contrasted with those generates by the AI assisted analysis.

*Manual analysis theme one: Authorship is human*

Across all the studied publisher policies, it became clear that authorship is seen as a uniquely human endeavour. While this is at first glance perhaps unsurprising, the way in which the theme is operationalised is of interest. Almost all of the publishers studied did not disallow the use of ChatGPT and LLMs, or other unspecified GenAI in order to assist with writing. It became clear that, either implicitly or explicitly, most publishers believed that despite their limitations, GenAI tools can make significant contributions to academic work. The example below from Thieme publishing demonstrates this point:

*AI tools such as ChatGPT can make scholarly contributions to papers. The use of generative AI tools should be properly documented in the Acknowledgements or Material and Methods sections.*

However, none of the publishers stated that LLMs or GenAI tools can be listed as authors. This is not because the models are not able to generate meaningful insight, content, analysis, or interpretation. Rather, the major focus is that such tools cannot take responsibility for the written work. The examples below from MIT Press and De Gruyter clearly demonstrate this point:

*The MIT Press does not allow artificial intelligence (AI) tools such as ChatGPT or large language models (LLMs) to be listed as authors of our publications. The emerging consensus of scholarly organizations, including the Committee on Publication Ethics, is that AI tools do not meet the requirements for authorship since they cannot assume ethical and legal responsibility for their work.*

*Please note that we do not accept papers that are generated by Artificial Intelligence (AI) or Machine Learning Tools [as authors] primarily because such tools cannot take responsibility for the submitted work and therefore cannot be considered as authors.*

*Manual analysis theme two: Policies are ambiguous*

The second most prevalent theme is ambiguity. Ambiguity in this sense does not cover whether it is unacceptable or acceptable to use a GenAI tool, as it is implied and explicitly stated in several policies that their use is allowed in the preparation of manuscripts for publication. However, whether and how the policies would be enforced is especially unclear, as is the permanence of the current policies. For example, the following extract from Elgar demonstrates that there are ‘verification tools’ that may be used in detecting GenAI-created text in manuscripts:

*The publisher reserves the right to verify the use of ChatGPT (or related platforms) and to reject manuscripts that violate this policy.*

While the above suggests that tests will be undertaken on writing to detect any GenAI text, there is no further information given about the verification process undertaken and whether it applies to all manuscripts or only those deemed suspicious. Nevertheless, this provides an ambiguous basis for policy enforcement and application. A similar sense of ambiguity is found in the permanence of many policies, as Elsevier, Springer, and Emerald demonstrate:

*Elsevier will monitor developments around generative AI and AI-assisted technologies and will adjust or refine this policy should it be appropriate. (Elsevier)*

*As we expect things to develop rapidly in this field in the near future, we will review this policy regularly and adapt it if necessary. (Springer)*

*Whilst industry standards will likely coalesce in the coming months, Emerald is adopting the following approach, as part of a watching brief on developments in the rapidly evolving AI space. (Emerald)*

This ambiguity recognises that the development of AI and LLMs is happening at an astounding pace, and thus, the current guidelines are subject to change as deemed appropriate. On the other hand, there is little explanation of when the review might be undertaken, and what developments might prompt changes to the policy. While the idea of ‘appropriacy’ and ‘necessity’ is mentioned, this leaves considerable room for interpretation. Emerald publishing suggests that a likely future scenario is the convergence of academic publishing policies and norms, as more about the applications of GenAI in academia are uncovered. Overall, this theme suggests that publishers have attempted to tentatively provide guidelines on the use of AI and LLMs in academic writing and publishing but with a high level of ambiguity and uncertainty.

*Manual analysis theme three: Transparency is vital*

From the analysis of publisher policies, the first core theme that can be outlined is that of authorship being a uniquely human endeavour based on the core concepts of agency, responsibility, and ownership of ideas. Second, while this is often conceptualised in concrete, unambiguous terminology, the actual operationalisation of AI and LLMs is allowable, and the policies of detection, enforcement, and use cases are highly open to interpretation and change. In essence, there is a sense from these two themes that both the rigidness of ownership and fluidity of rules can operate together.

The third theme is a similar all-encompassing requirement: transparency. While there are no prohibitions on using GenAI tools in the writing process in any of the policies examined, there are commitments to principles everywhere, and transparency forms one of the most frequently cited principles in the data. The examples below from COPE, MDPI, and Cambridge University press demonstrate the commitment to transparency as a cornerstone of policies regarding AI tools:

*Authors who use AI tools in the writing of a manuscript, production of images or graphical elements of the paper, or in the collection and analysis of data, must be transparent in disclosing in the Materials and Methods (or similar section) of the paper how the AI tool was used and which tool was used.* – COPE

*Furthermore, authors are required to be transparent about the use of these tools and disclose details of how the AI tool was used within the “Materials and Methods” section*. – MDPI

*Any use of AI must not breach Cambridge’s plagiarism policy. Scholarly works must be the author’s own, and not present others’ ideas, data, words or other material without adequate citation and transparent referencing.* – Cambridge University Press

In the extract from COPE, it is clear that the expectation is to list the use of such tools in the Materials and Methods section of the manuscript, which is also found in the requirements of MDPI. This statement suggests that an LLM or GenAI tool is an aid to the research process, rather than an author. This is congruent with the perspective that AI tools cannot fulfil the role of an author, but does not fully capture the fact that such tools can generate novel text. Furthermore, the extent to which AI tools must be declared is not clear. AI tools can assist in finding resources for literature reviews, summarising existing studies, or providing help with study design or creation. As such, there are gaps in the comprehensiveness of AI policy statements for several publishers, and transparency is considered a core fundamental value. However, it is assumed that authors are aware of the exact limits of transparency in a rapidly evolving AI-driven research landscape which will likely not always be the case.

This is an important aspect of the theme because it identifies a unique aspect of LLMs and GenAI tools, which seems to be true at present. When posed with question ‘Can you take responsibility for a written work?’, ChatGPT 3.5 (24 ^th^ July, 2023) provided the following answer:

*As an AI language model, I don’t have personal agency or the ability to take responsibility for any written work. Any text generated by me is a result of algorithms and training data up until my knowledge cutoff date in September 2021. The responsibility for the content and its usage lies with the user, who inputs the text and decides how to utilise it.*

Therefore, it seems that the fact that textual output produced by LLMs is not ‘considered’ and does not go through a reflective or fact-checking process is one of the key aspects that leads it to be unable to fulfil authorship criteria. However, this has not been explicitly discussed in many publisher policies. Rather, ‘not able to fulfil authorship criteria’ is used as a general sentence, but without a detailed explanation. For those who are unfamiliar with how tools such as LLMs work and why they are unable to produce innovative, novel insights or generate new knowledge, this may be confusing. Although in an ideal world, those considering submission to a scholarly journal would complete due diligence on such matters, this is not always the case.

In general, this theme indicates that publisher policies identify the unique humanity of authoring works. This means not just producing texts that are coherent, informative, or sound highly polished and professional but also the agency and capacity to understand the potential ethical, legal, and social ramifications of knowledge production.

Across the categories of publishers, including scholarly publishers, university publishing houses, general book publishers, newspapers, and NGOs, no significant differences in approach were noted.

### Results section two: GenAI supported thematic analysis

In order to conduct the GenAI assisted thematic analysis, R1 began by accessing OpenAI’s ChatGPT interface, using the paid subscription option ‘ ChatGPT Plus’ to access the more advanced underlying LLM, GPT-4. Several modes were previously available using this subscription, including ‘Advanced Data Analysis (previously named ‘Code Interpreter’), which has now been combined into one overall ‘GPT-4’ model. Both the default mode and Code Interpreter/Advanced data analysis mode were used to identify themes at two separate points in time: July 27 ^th^ and September 11 ^th^ 2023. This was done to demonstrate the continuously advancing nature of the software, highlighting the importance of authors being specific about their use of GenAI tools in the research process. The nature of the analysis driven by GenAI resulted in the output of completed themes by the software when prompted. This is an important aspect to recognise when using a GenAI tool to assist with thematic analysis: some steps are not immediately accessible or visible to the researcher, or may be skipped over altogether.

The process began with R1 providing the collected publisher data to the LLM structured using the following prompt:

*Perform an inductive thematic analysis on a provided dataset of recently created policies related to generative AI from various publishers. The dataset is structured with the columns ‘policy number’, ‘name of publisher’, ‘publisher category’, and ‘policy’. Compare the differing approaches taken by these publishers based on their categories. The results should be provided in a markdown table with at least two specific examples drawn from the policies to illustrate the themes created. Additionally, provide a text summary of the results suitable for inclusion in an academic journal #here is the table.*

Table 1 shows the themes identified in the initial analyses conducted in July, 2023.

**Table 1.** July 2023 AI thematic analysis.

Default mode		Code interpreter	
Theme	Description	Theme	Description
**Authorship**	AI tools do not qualify for authorship.	**Use of AI Tools**	The policies in this theme primarily address the use of AI in the context of authoring and publishing. They highlight the integration of AI technologies in generating and refining content.
**Accountability**	Authors are accountable for their work, including any content produced by AI tools.	**AI Authorship**	These policies grapple with the implications of AI tools for authorship, addressing issues of intellectual property and responsibility.
**Disclosure**	Use of AI tools must be disclosed.	**Access and Regulation**	This theme is characterized by policies focused on access to and regulation of AI tools and generated content.
**Integrity**	Use of AI tools should not compromise research integrity.		
**Policy Evolution**	Policies on AI use are subject to change as the field evolves.		

Multiple errors were produced during the attempted thematic analysis using the code interpreter mode. This software had only been released for under a week and struggled to handle the Excel data file provided. The themes were also not produced in the same style as the standard model, with simple descriptions absent from the tables. The thematic analysis was also noticeably more limited than that produced by the default GPT4 model, with only three high-level themes being identified.

Table 2 shows the themes identified in the second analysis conducted in September 2023.

**Table 2.** September 2023 AI thematic analysis.

Default mode		Advanced data analysis	
**Accountability for Manuscript Integrity**	Emphasizes that authors are solely responsible for the entire content of the manuscript, including parts generated with AI.	**Accountability and Authorship**	Emphasizes that AI cannot be credited as an author due to a lack of accountability.
**AI Tool Transparency**	Requires authors to disclose the use of AI tools in the paper, chapter, or case study. Any method, data collection, or image generation done using AI should be transparently mentioned.	**Transparency and Disclosure**	Requires authors to disclose the use of AI tools in their work.
**Role in Authorship**	Consensus among publishers that AI tools do not meet the criteria to be listed as authors.	**Scope and Limitations**	Specifies what AI can and cannot be used for within the research and writing process.
**Ethical Considerations**	Mentions or refers to ethical guidelines and frameworks like COPE, focusing on the need for AI tools to meet ethical standards.	**Ethical Considerations**	Addresses ethical considerations such as plagiarism and research integrity.
**Impact of Evolving AI**	Acknowledges the potential for AI technology to evolve and impact future policies.		
**Complete Rejection of AI Tools**	An outright ban on the use of AI tools in the submission process, indicating a zero-tolerance policy toward AI-assisted content.		

The analysis of publisher policies undertaken in July and September reveals interesting distinctions between the default and advanced data analysis modes available in ChatGPT Plus. In the default mode, the identified themes remained largely consistent, with only one new theme, ‘Complete Rejection of AI Tools’, which emerged in the September analysis. The theme ‘Impact of Evolving AI’ subtly diverged from ‘Policy Evolution,’ indicating a minor refinement rather than a major shift. However, the advanced data analysis tool demonstrated major differences compared to the original analysis, which could point to more rapid advancements or refinements in this beta tool. Rather than increasing the intricacy of the thematic structure, it consolidated themes like “Accountability” and “Authorship” into a singular construct, thus reducing the overall number of themes. Despite operating on the same underlying dataset, these two modes of analysis yielded different thematic outcomes, not due to a changing landscape, but rather due to their respective analytical frameworks. No differences between the different categories of publishers were discovered in any of the analyses.

When all themes created by the GenAI tool were compared, similarities emerged that enabled R1 to create the final AI-assisted themes, a process carried out by manually grouping the previous themes that had emerged and asking for ChatGPT (default model) to create a revised name and description for the combined themes. The names and descriptions were then manually edited. The final themes for the AI assisted thematic analysis are shown in Table 3.

**Table 3.** Development of AI assisted themes.

July 27 ^th^ analysis		September 11 ^th^ analysis		Final AI assisted analysis theme	
Default	Code interpreter	Default	Advanced data analysis	AI Assisted theme	Description
AI Cannot Author	AI Authorship	Role in Authorship	Accountability and Authorship	**Authorship Constraints**	AI tools ineligible for authorship due to lack of accountability and legal standing.
Accountability	Use of AI Tools	Accountability for Manuscript Integrity	_	**Human Accountability**	Authors solely responsible for AI-assisted or generated content.
Disclosure	_	AI Tool Transparency	Transparency and Disclosure	**Transparency in AI Utilisation**	Mandatory disclosure of AI tool usage in manuscripts.
Integrity	Access and Regulation	Ethical Considerations	Ethical Considerations	**Ethical and Integrity Concerns**	Addresses ethical and integrity issues in AI-assisted research.
Policy Evolution	_	Impact of Evolving AI	Evolving Policies	**Adaptability of Policies**	Indicates evolving policies due to rapid AI advancements.
_	_	Complete Rejection of AI Tools	Scope and Limitations	**Limitations and Prohibitions**	Specifies restrictions or outright rejections of AI-assisted content.

The final selected AI-assisted themes, based on the four analyses requested from ChatGPT, are discussed below. Examples of quotes from policies representing the themes were requested in all stages of the data analysis, however, despite multiple prompting attempts over the different modes of analysis, it was not possible to obtain fully accurate examples from ChatGPT, with quotes frequently misattributed to other publishers, condensed, or simply fabricated. These LLM hallucinations demonstrate that, although GenAI tools can offer support in research activities, their results must be carefully checked to ensure accuracy. All quotes from the policies shown below were manually selected and/or verified.

*AI assisted analysis theme one: Authorship constraints*

The dominant theme from the analysis emphasises that AI tools lack the capability to qualify for authorship, primarily because of the absence of accountability and legal standing. Examples from Cambridge University Press and Palgrave MacMillan/St Martin’s Press. corroborate this stance: “ *AI does not meet the Cambridge requirements for authorship, given the need for accountability*,” and “ *Large Language Models (LLMs), such as ChatGPT, do not currently satisfy our authorship criteria.*” Interestingly, the focus here seems less on extolling human qualities of responsibility (as identified in the manual analysis) and more on delineating the limitations inherent to AI in the context of authorship.

*AI assisted analysis theme two: Human accountability*

The second theme in the assisted analysis is Human Accountability, specifically the claim that authors are accountable for all the work they produce and submit, including any content produced by AI tools. This was supported in the initial AI results by a quote from MIT Press: “ *Authors are fully responsible for the content of their manuscripts, including any portions produced by AI tools*” and Springer: “ *Notably an attribution of authorship carries with it accountability for the work, which cannot be effectively applied to LLMs*”, and SSRN “ *Authors are ultimately responsible and accountable for the contents of their work*” amongst others.

This theme is similar to the first theme identified in the manual analysis of ‘authorship is human’ because of the unique human ability to take responsibility for the content of the work. In this sense, although the assisted analysis identified the same element in the policies, this was separated out into an entire theme itself, while R2 included it more holistically under the category of authorship being fundamentally human.

### AI assisted analysis theme three: Transparency in AI utilisation

The third theme identified in the assisted analysis is related to the transparent disclosure of AI tools when submitting a manuscript for publication. Examples include Edward Elgar’s guidelines: “ *If authors choose to use AI tools to assist in their research, they must disclose this in the manuscript*,” and Cambridge University Press’s requirement that “ *AI use must be declared and clearly explained.*” The publishers seem to balance acknowledgement of AI’s utility with its ineligibility for authorship, aligning with the manual theme “Transparency is Vital.”

Across almost all publishers, it was found that there was a requirement to disclose the use of AI tools, reflecting an evolving consensus that simultaneously seems to acknowledge the use of AI tools and their ability to make genuine contributions, while equally denying that they fit the category of an author. This is similar to the theme identified in the manual analysis of ‘Transparency is Vital’. Again, the analysis indicates that there is a high degree of consistency in the relevance of the themes that have been identified separately, yet also demonstrates that there are nuances in the classification of themes as belonging to a broader category (as with manual analysis) or that they constitute discrete themes in themselves (assisted analysis).

### AI assisted analysis theme four: Ethical and integrity concerns

The fourth theme identified was that the use of AI tools should not compromise research integrity. This was supported by examples from Elsevier Science Ltd: “ *Authors are accountable for ensuring that questions related to the accuracy or integrity of any part of the work are appropriately investigated and resolved”,* and Wiley-Blackwell: “ *The author is fully responsible for the accuracy of any information provided by the tool and for correctly referencing any supporting work on which that information depends*”.

Interestingly, this was not identified as a theme in the manual analysis. Following the discussion and comparison of data coding and analysis, it was identified that R2 felt that the concept of integrity was related more wholly to the theme of transparency and authorship being uniquely human. Following this, if an author was not transparent and did not take responsibility for their work, then this by itself would represent a lack of integrity, and that integrity formed a larger ‘macro theme’ of the entire policy data rather than just the data referencing AI. In this sense, R2’s familiarity with the overall policies may have had an effect, whereas the AI-assisted analysis was based solely on data provided by the extracted AI policy.

### AI assisted analysis theme five: Adaptability of policies

The fifth theme discusses the dynamism inherent in AI policies, acknowledging the speed at which AI technology evolves, and how policies will have to evolve alongside these changes. Examples from Edward Elgar and Emerald indicate keen awareness of this fluid landscape. Edward Elgar remarks, “ *As this is a fast-moving area, this policy is subject to change*” while Emerald notes, “ *Emerald is adopting the following approach, as part of a watching brief on developments in the rapidly evolving AI space*”. This parallels the manual theme of ‘ambiguity’, although the focus is more on the adaptability of policies rather than on their indeterminate nature.

### AI assisted analysis theme six: Limitations and prohibitions

The sixth and final theme outlines the limits, boundaries, and restrictions imposed on the use of AI tools, sometimes extending outright prohibitions. The sixth theme elucidates the stringent restrictions on the use of AI tools in academic settings. Edward Elgar’s policy is particularly explicit: “ *The use of AI tools such as ChatGPT (or related platforms) to generate substantive content, such as the analysis of data or the development of written arguments, is not permitted.*” This sets a clear boundary on the roles that AI tools can play in academic research, underlining the limitations that publishers believe are necessary to ensure ethical integrity. SSRN also emphasises that “ *Authors should only use AI technologies to improve the readability and language of their work and not to replace key researcher/author tasks”.* In the broader publishing ecosystem, other non-academic publishers appear to go even further than this, as exemplified by Clarkesworld’s firm stance: “We will not consider any submissions written, developed, or assisted by these tools. Attempting to submit these works may result in being banned from submitting works in the future”.

### Revision of themes

The themes identified by the AI-assisted analysis as well as from the manual analysis were then used as a basis for further investigation and reflection to support the development of a final set of themes which both authors believed adequately represented the data. Table 4 summarises the development of the final identified themes.

**Table 4.** Finalised themes.

Manual analysis	Description	AI-Assisted analysis	Description	Final themes	Description
**Authorship is human**	Publisher policies universally designate authorship as a human endeavour, acknowledging GenAI tools like ChatGPT can aid but not assume responsibility in academic writing.	**Authorship Constraints**	AI tools ineligible for authorship due to lack of accountability and legal standing.	**Human-Exclusive Authorship**	AI tools can assist in the writing and research process but cannot be granted authorship due to their inability to assume responsibility or ethical obligations. Human authorship is viewed as unique and irreplaceable in this context.
		**Human Accountability**	Authors solely responsible for AI-assisted or generated content.	**Author Accountability**	While GenAI tools can offer support in the research process, authors themselves are fundamentally responsible for their work.
**Transparency is vital**	Transparency about the use of AI tools is a recurrent principle in publisher policies, although the exact requirements for disclosure remain vague.	**Transparency in AI Utilisation**	Mandatory disclosure of AI tool usage in manuscripts.	**Disclosure and Transparency**	Disclosure of AI tool usage is a universally accepted requirement, but the degree and format of that disclosure are not consistently defined across different publishers. Authors should provide detail on how the tools were used, as opposed to just disclosing them.
		**Ethical and Integrity Concerns**	Addresses ethical and integrity issues in AI-assisted research.	**Research Integrity**	The use of AI tools should align with the ethical standards and integrity of the research, ensuring that their application does not compromise the quality or veracity of the academic work.
**Policies are ambiguous**	Publisher guidelines on AI usage are often unclear in terms of enforcement and permanence, reflecting the rapid pace of AI development.	**Adaptability of Policies**	Indicates evolving policies due to rapid AI advancements.	**Fluid Policy Landscape**	Policies concerning the use of AI in academic publishing are in a state of flux, adapting to both technological advancements and the evolving ethics of its implications.
		**Limitations and Prohibitions**	Specifies restrictions or outright rejections of AI-assisted content.	**Constraints and Exclusions**	Policies outline specific conditions and explicit prohibitions on the use of AI-assisted tools in areas of the research process, ranging from limitations in specific contexts to outright exclusion.

## Discussion

The final six themes were developed and labelled through a collaborative effort involving both AI-assisted and manual coding approaches. This process entailed using ChatGPT to assist in theme identification and subsequent discussions between the two authors to refine and elaborate on these themes, along with cross-checking and contrasting the data collected using the two different methods (manual *versus.* AI-supported). This resulted in a set of final themes that encapsulate the complexities and nuances associated with the use of AI in academic publishing, with three themes being jointly identified by both the GenAI analysis and the manual analysis, and the three additional themes identified by the AI analysis being retained but expanded and modified slightly. Overall, this methodology seemed productive. While there are risks in terms of using such software to coordinate and undertake analysis, in our experience, human intervention, oversight, and a robust triangulation process with manual coding and analysis helped eliminate the risk of errors.

The major benefit of the AI-assisted theme development was the broader range of themes that emerged, including some that appeared overly obvious to a human researcher. However, on reflection, the researchers agreed that the AI appeared to capture additional subtle themes present in the data. This said, the data used for this analysis are straightforward, simple, and less complex than, for example, interview data which spans several topics and are longer in duration/text length. Consequently, further studies are needed to examine the differences that might be generated from dual analyses in this manner. We suggest that in the future, in similar studies, it is beneficial to adopt this two-system approach, in which manual ‘traditional’ analysis is conducted, and AI-generated analysis is then used to further enrich and triangulate findings.

One of the major areas of interest in the results was the striking similarity between the themes identified by both methods. The most clearly related of these is the notion of ‘Human-Exclusive Authorship’, which combines the ‘Authorship Constraints’ theme from the AI-assisted analysis and the ‘Authorship is Human’ theme from the manual analysis. Both analyses concur that authorship in academic contexts is a uniquely human endeavour, and this suggests a high degree of certainty in the validity of our themes and the consistency of the data. In summarising the content of this theme, the consensus that appeared to emerge from the policies is that AI tools, including Generative AI technologies such as ChatGPT, can assist in various aspects of research and writing but fall short of assuming the ethical and legal responsibilities that come with authorship. This reflects a broader consensus in the academic community that acknowledges the supportive role of AI tools while underscoring the irreplaceable value of human expertise and ethical judgement.

Another overlap was clear between the themes of ‘Transparency in AI Utilisation’ and ‘Transparency is Vital’. Both analyses underscored the importance of transparency when using AI tools in academic work. However, the AI-assisted analysis focused more on the need for disclosure as a mandate, whereas manual coding highlighted the principle of transparency. The final theme of ‘Transparency and Disclosure’ encapsulates this nuance, emphasising not only the necessity of disclosure, but also its variable implementation across different publishers. From this, we can see that, while some minor differences occur in the micro-focus of the themes depending on the manual or assisted analysis, the general idea of transparency was picked up by both researchers. The necessity of transparency when using GenAI tools in academic research appears in almost every policy examined. Transparency in this case is not merely a procedural requirement but a fundamental ethical principle that upholds the integrity of academic scholarship and serves multiple purposes. First, it allows for verification of the methods employed, which is crucial for the replicability and validity of the research. Second, it ensures that the contributions of the AI tools are appropriately acknowledged, thereby providing a clear demarcation between human and machine contributions. This is particularly important given that GenAI tools can perform a wide array of functions, ranging from data collection and analysis to generating text for literature reviews. Failure to disclose the extent of AI involvement could inadvertently mislead readers and obfuscate the human intellectual labour involved, thereby undermining the ethical foundations of scholarly work.

The themes “Policies are Ambiguous” from manual coding and “Evolving Policies” from the AI-assisted analysis converge into the final theme of “Fluid Policy Landscape.” This theme captures the dynamic and likely ever-changing nature of policies concerning the use of AI in academic publishing. Both analyses recognise that these policies are not static; they are subject to change in response to the rapid advancements in AI technology and shifts in ethical understanding and acceptance of these tools as they become more widely known and used in the academic community. This theme encapsulates this complexity, underscoring that, while there is a need for guidelines, these policies are inherently fluid and need to be responsive to the rapidly evolving landscape of AI technologies and their ethical implications.

In considering the final theme of ‘Constraints and Exclusions’, we find this particularly salient in the context of the ongoing discourse around the legitimacy and ethicality of AI utilisation in academic work that was highlighted by the fifth theme. This theme reflects the often stringent guidelines and explicit prohibitions found in the policies of various academic publishers. This theme highlights the field’s cautious approach to incorporating AI into academic processes, especially in generating a significant proportion of intellectual output, thus closely aligning with other themes that emphasise human responsibility and ethical considerations. This theme illuminates the existence of broader concern, transcending academic circles about the capabilities and limitations of AI in content creation. While the policies are not uniform across all publishers, the overall tenor of the discussion seems to be one of caution, placing the onus on human researchers to navigate the evolving landscape. Therefore, the theme ‘Constraints and Exclusions’ serves as a cautionary note, emphasising that, while AI technologies offer vast potential for enhancing academic work, they are not without their limitations and must be used judiciously.

These themes offer an in-depth understanding of the complexities surrounding the use of AI in academic publishing. They reflect the collective insights gained from leveraging both AI and human analytical capabilities, thereby providing a robust framework for future discussions and research in this area.

### Limitations and implications

This research has provided insights into the current systems of thought regarding the use of AI and academic publishing. There is no doubt that this is a complicated, evolving issue, but at the same time, it seems that there is a high degree of consistency among academic publishers about the role that AI tools are currently fulfilling. Equally, it seems that the core principles of academic integrity, authorship, and responsibility have not undergone any form of dramatic change. Rather, it seems that the current view is that such applications are another asset in the toolbox of academic scholars. In addition to the ease of use of these tools, there are also arguments that they may contribute to the democratisation of research and scientific knowledge. AI tools can, for example, benefit non-native speakers of English who conduct research and are under pressure to publish in English-medium journals by providing rapid support and feedback on manuscripts or enable new scholars and students who are engaging in academic publishing for the first time to receive additional guidance and support on writing for publication. Students engaged in postgraduate or doctoral studies may also use these tools to help develop and refine their ideas, presentations, and proposals to enhance their chances of success in the competitive academic field. For authors who are experienced and confident writers or composing text in the first language, there are still opportunities to improve the logic of expression, coherence, style, and quickly change formatting to enable submission to a target journal. Therefore, it is encouraging to see that scholarly publishers have not generally taken the path of prohibition or reactionary banning of AI in the research and writing process.

However, alongside these positive implications are challenges that the academic community must navigate. One of these is the phenomenon known as ‘stochastic parroting’, where AI tools may simply repeat training data ( Li, 2023) or ‘hallucinations’ where it may inadvertently produce misleading or incorrect information ( Bender *et al.*, 2021); a by-product of their training data and algorithms. This raises questions about the reliability and integrity of AI-generated content, mandating extra scrutiny from authors (as demonstrated in the AI analyses insistence on creating false quotes from the policies). The issue compounds when we consider the potential for AI tools to produce voluminous yet generic output, adding an additional layer of complexity to the review and editing process in journal production. Editors and reviewers may find themselves burdened by the task of sifting through and filtering this added volume, questioning the originality and contribution of AI-assisted manuscripts. Moreover, the ease of generating academic content using AI tools can be a double-edged sword. On the one hand, these tools may accelerate the research process by supporting in cognitive offloading ( Dawson, 2020; Perkins *et al.*, 2023); the process of reducing the cognitive demands of a task ( Risko & Gilbert, 2016). However, this must be balanced by the risks of authors committing research misconduct by providing false information or disguising authorship. This underscores the need for robust policy frameworks to guide ethical AI use in academic research. For example, policies should clarify the extent to which AI tools need to be disclosed in academic work, as found in our analysis of various publishers’ stances on AI authorship and accountability. This can be conducted within the framework of cognitive offloading if any use of GenAI tools is explained adequately and transparently. Another limitation is the current inability of AI to match human scholarship in terms of nuanced contextual understanding and interpretative abilities. As our thematic analysis revealed, both human and AI-assisted reviews emphasise that, while AI tools can be highly beneficial for specific tasks, they cannot replace human intuition and judgement. This is particularly significant in the realms of hypothesis formation and qualitative interpretation, areas in which human cognition currently has an insurmountable edge.

Regarding the limitations of the study itself, it is crucial to acknowledge that our inductive thematic analysis was confined to a limited set of publisher policies available at the time of the research. The rapidly evolving nature of AI technology and its applications in academia means that the landscape is fluid and policies may have already changed. Similarly, we focused our attention on academic publishing in English, and among flagship and major publishing houses. This also comes at the expense of focusing on non-academic publishing, such as fiction and general readership texts, and publishing guidelines for publications produced by professional and governmental organisations. Further research in this area would be useful to expand the field of study on the use of generative AI in publication at large. This study also relied on a combination of AI-assisted and human analysis, each with its own set of biases and limitations. For example, the use of a single AI tool leaves the door open to software-specific errors or idiosyncratic features; however, this was minimised by using four separate analyses over two time periods. Future research should aim to mediate this by conducting an analysis using a range of GenAI tools and comparing the results. Although the GenAI analysis was capable of processing large volumes of data in a short time span, it may have missed nuanced interpretations that a human researcher could capture. Conversely, human analysis, although in-depth, was limited by the constraints of time and cognitive load, which AI does not face. Furthermore, the study did not empirically test the effectiveness or reliability of AI tools in academic research, which is a potential avenue for further research. Therefore, the findings should be considered a snapshot of the complex and evolving interplay between AI technology and academic publishing.

## Conclusion

In conclusion, the multifaceted roles and implications of AI tools in academic research warrant a more inclusive and forward-thinking approach than is currently demonstrated. As our study shows, there is broad consensus among publishers that authorship in academic contexts remains a uniquely human endeavour. Human authors are irreplaceable in their capacity to assume ethical and legal responsibilities, and are qualities that current AI technologies cannot replicate. However, this recognition should not overshadow the valuable supportive roles that GenAI tools can fulfil. From text generation and data analysis to linguistic translation and textual editing, these technologies can democratise access to academic publishing and facilitate a more inclusive scholarly ecosystem, mediating inequalities that occur in terms of linguistic ability and experience in publishing academic work.

The themes identified in our study highlight the complexities and nuances of integrating AI into academic research but encouragingly suggest that publishers are aware of the significant benefits that scholars can reap from using such tools in a transparent and ethical manner. They underscore the necessity for a balanced approach that is both open to AI’s potential and vigilant of its ethical challenges. Such an approach calls for policies that are not merely reactionary, but are formulated based on a nuanced understanding of AI’s capabilities and limitations. An overly conservative stance toward AI integration in academic settings risks stifling innovation and could result in guidelines that are more obstructive than facilitative, as seen in the more severe restrictions imposed by some publishers.

Moreover, the fluid nature of AI technologies necessitates that publisher policies are agile and adaptive. Our findings indicate that, while there is a consensus on the non-qualification of AI for authorship and the imperative of accountability and transparency, there is also a shared understanding that these stances will need to evolve in tandem with technological advancements. Dialogues around the use of AI in academic research are intricate and evolving, and while the core values of human authorship and accountability remain constant, there is increasing acknowledgement of the supportive roles that AI can play in academic research. As computational power grows and societal acceptance of AI becomes the norm, it is likely that shifts in how GenAI supported content is perceived will take place. Therefore, it becomes crucial for academic institutions and publishers to focus on developing informed and flexible guidelines that consider changes in technology and societal perceptions, while safeguarding the principles of academic integrity. Such a holistic approach will not only serve to uphold the ethical foundations of academic research but also pave the way for innovative practices that capitalise on the synergies between human intellect and artificial intelligence, as demonstrated in this work.

### AI usage disclaimer

This study used Generative AI tools to analyse data, create preliminary themes, produce draft text, and revise wording throughout the production of the manuscript. Multiple modes of ChatGPT over different time periods were used, with all modes using the underlying GPT-4 foundation model. The authors reviewed, edited, and take responsibility for all outputs of the tools used in this study.

## Data Availability

Figshare: Generative AI policies for academic publishers https://doi.org/10.6084/m9.figshare.24124860.v1 ( Perkins, 2023b). This project contains the following underlying data:
• Gen AI policies Academic Publisher.xlsx. (All data related to AI policies including full policies as downloaded and URLs). The data is available under the terms of the CC-BY 4.0 license.

## Footnotes

^1^
‘In all content submitted to SSRN, authors should only use AI technologies to improve the readability and language of their work and not to replace key researcher/author tasks such as producing insights or theories, analyzing and interpreting data or drawing conclusions and presenting viewpoints.’( Elsevier, 2023).

^2^
‘The use of AI tools such as ChatGPT (or related platforms) to generate substantive content, such as the analysis of data or the development of written arguments, is not permitted’(Edward Elgar Publishing, n.d.).

## References

[ref1] Ahmed I Jeon G Piccialli F : From Artificial Intelligence to Explainable Artificial Intelligence in Industry 4.0: A Survey on What, How, and Where. *IEEE Trans. Industr. Inform.* 2022;18(8):5031–5042. 10.1109/TII.2022.3146552

[ref2] Ahmed S Alshater MM Ammari AE : Artificial intelligence and machine learning in finance: A bibliometric review. *Res. Int. Bus. Financ.* 2022;61:101646. 10.1016/j.ribaf.2022.101646

[ref3] Anderson N Belavy DL Perle SM : AI did not write this manuscript, or did it? Can we trick the AI text detector into generated texts? The potential future of ChatGPT and AI in Sports & Exercise Medicine manuscript generation. *BMJ Open Sport Exerc. Med.* 2023;9(1):e001568. 10.1136/bmjsem-2023-001568 36816423 PMC9936276

[ref4] Baidoo-Anu D Owusu Ansah L : *Education in the Era of Generative Artificial Intelligence (AI): Understanding the Potential Benefits of ChatGPT in Promoting Teaching and Learning*(SSRN Scholarly Paper 4337484).2023. 10.2139/ssrn.4337484

[ref5] Bao J Xie Q : Artificial intelligence in animal farming: A systematic literature review. *J. Clean. Prod.* 2022;331:129956. 10.1016/j.jclepro.2021.129956

[ref6] Beallslist.net: Beall’s List – of Potential Predatory Journals and Publishers. 2021. Retrieved 12 October 2023. Reference Source

[ref7] Bender EM Gebru T McMillan-Major A : On the Dangers of Stochastic Parrots: Can Language Models Be Too Big? *Proceedings of the 2021 ACM Conference on Fairness, Accountability, and Transparency.* 2021; pp.610–623. 10.1145/3442188.3445922

[ref8] Biswas SS : Role of Chat GPT in Public Health. *Ann. Biomed. Eng.* 2023;51(5):868–869. 10.1007/s10439-023-03172-7 36920578

[ref69] Boyatzis RE : *Transforming Qualitative Information: Thematic Analysis and Code Development.* SAGE;1998.

[ref9] Braun V Clarke V : Using thematic analysis in psychology. *Qual. Res. Psychol.* 2006;3(2):77–101. 10.1191/1478088706qp063oa

[ref10] CAIS: Statement on AI Risk|CAIS. 2023. Reference Source

[ref60] Cole S Kiss E : What Can we do about Student Cheating. About Campus. 2000;5(2):5–12. 10.1177/108648220000500203

[ref11] Cotton DR Cotton PA Shipway JR : Chatting and cheating: Ensuring academic integrity in the era of ChatGPT. *Innov. Educ. Teach. Int.* 2023;1–12. 10.1080/14703297.2023.2190148

[ref12] Crawford J Cowling M Ashton-Hay S : Artificial Intelligence and Authorship Editor Policy: ChatGPT, Bard Bing AI, and beyond. *J. Univ. Teach. Learn. Pract.* 2023;20(5). 10.53761/1.20.5.01

[ref13] Curtis GJ : Trends in plagiarism and cheating prevalence: 1990-2020 and beyond. Rettinger D Bertram Gallant T , editors. *Cheating academic integrity.* Jossey-Bass;2022; pp.11–44. Reference Source

[ref65] Dawson P : Cognitive Offloading and Assessment. Bearman M Dawson P Ajjawi R editors. Re-imagining University Assessment in a Digital World. Springer International Publishing;2020; pp.37–48. 10.1007/978-3-030-41956-1_4

[ref14] Debrah C Chan APC Darko A : Artificial intelligence in green building. *Autom. Constr.* 2022;137:104192. 10.1016/j.autcon.2022.104192

[ref62] Eaton SE : The Academic Integrity Technological Arms Race and its Impact on Learning, Teaching, and Assessment. *Canadian Journal of Learning and Technology/Revue Canadienne de l’apprentissage et de La Technologie.* 2022;48(2):1–9. 10.21432/cjlt28388

[ref16] Edward Elgar Publishing: *Elgar copyright policy: Your guide to the essentials.* Edward Elgar Publishing;n.d.Retrieved 8 September 2023. Reference Source

[ref17] Elias J : *Google CEO Sundar Pichai warns society to brace for impact of A.I. acceleration, says ‘it’s not for a company to decide’.* CNBC;2023, April 17. Reference Source

[ref18] Elsevier: What is SSRN’s policy on the use of generative AI and AI-assisted technologies in papers posted at SSRN? - SSRN Support Center. 2023, February 20. Reference Source

[ref19] European Parliament: EU AI Act: First regulation on artificial intelligence|News|European Parliament. 2023, August 6. Reference Source

[ref20] Gao CA Howard FM Markov NS : Comparing scientific abstracts generated by ChatGPT to original abstracts using an artificial intelligence output detector, plagiarism detector, and blinded human reviewers. 2022. 10.1101/2022.12.23.521610

[ref21] Garcia MB : Using AI Tools in Writing Peer Review Reports: Should Academic Journals Embrace the Use of ChatGPT? *Ann. Biomed. Eng.* 2023. 10.1007/s10439-023-03299-7 37368125

[ref22] Hammad M : The Impact of Artificial Intelligence (AI) Programs on Writing Scientific Research. *Ann. Biomed. Eng.* 2023;51(3):459–460. 10.1007/s10439-023-03140-1 36637603

[ref23] Henley J : German tabloid Bild cuts 200 jobs and says some roles will be replaced by AI. *The Guardian.* 2023, June 20. Reference Source

[ref24] Hu G : Challenges for enforcing editorial policies on AI-generated papers. *Account. Res.* 2023;1–3. 10.1080/08989621.2023.2184262 36840450

[ref25] Hutson M : Could AI help you to write your next paper? *Nature.* 2022;611(7934):192–193. 10.1038/d41586-022-03479-w 36316468

[ref26] Jabotinsky HY Sarel R : Co-authoring with an AI? Ethical Dilemmas and Artificial Intelligence. 2022. 10.2139/ssrn.4303959

[ref27] Jiang Y Li X Luo H : Quo vadis artificial intelligence? *Discov. Artif. Intell.* 2022;2(1):4. 10.1007/s44163-022-00022-8

[ref28] Johnson J : Artificial intelligence & future warfare: Implications for international security. *Def. Secur. Anal.* 2019;35(2):147–169. 10.1080/14751798.2019.1600800

[ref29] King MR : The Future of AI in Medicine: A Perspective from a Chatbot. *Ann. Biomed. Eng.* 2023;51(2):291–295. 10.1007/s10439-022-03121-w 36572824

[ref30] Kumar Y Koul A Singla R : Artificial intelligence in disease diagnosis: A systematic literature review, synthesizing framework and future research agenda. *J. Ambient. Intell. Humaniz. Comput.* 2023;14(7):8459–8486. 10.1007/s12652-021-03612-z 35039756 PMC8754556

[ref31] Lee JY : Can an artificial intelligence chatbot be the author of a scholarly article? *J. Educ. Eval. Health Prof.* 2023;20. 10.3352/jeehp.2023.20.6 36842449 PMC10033224

[ref32] Li Z : The Dark Side of ChatGPT: Legal and Ethical Challenges from Stochastic Parrots and Hallucination (arXiv:2304.14347). arXiv. 2023. Reference Source

[ref33] Liebrenz M Schleifer R Buadze A : Generating scholarly content with ChatGPT: Ethical challenges for medical publishing. *Lancet Digit. Health.* 2023;5(3):e105–e106. 10.1016/S2589-7500(23)00019-5 36754725

[ref34] Lund BD Wang T Mannuru NR : ChatGPT and a new academic reality: Artificial Intelligence-written research papers and the ethics of the large language models in scholarly publishing. *J. Assoc. Inf. Sci. Technol.* 2023;74(5):570–581. 10.1002/asi.24750

[ref61] Mortati J Carmel E : Can We Prevent a Technological Arms Race in University Student Cheating? Computer. 2021;54(10):90–94. 10.1109/MC.2021.3099043

[ref64] Nguyen-Trung K Saeri AK Kaufman S : Applying ChatGPT and AI-powered tools to accelerate evidence reviews. 2023. Reference Source

[ref35] Nishikawa-Pacher A : Who are the 100 largest scientific publishers by journal count? A webscraping approach. *J. Doc.* 2022;78(7):450–463. 10.1108/JD-04-2022-0083

[ref59] Nowell LS Norris JM White DE : Thematic analysis: Striving to meet the trustworthiness criteria. *Int. J. Qual. Methods.* 2017;16(1). 10.1177/16094069177338

[ref36] OpenAI: GPT-4 Technical Report (arXiv:2303.08774). arXiv. 2023a. Reference Source

[ref37] OpenAI: Introducing ChatGPT Enterprise. 2023b, August 28. Reference Source

[ref38] Patel SB Lam K : ChatGPT: The future of discharge summaries? *Lancet Digit. Health.* 2023;5(3):e107–e108. 10.1016/S2589-7500(23)00021-3 36754724

[ref39] Pavlik JV : Collaborating With ChatGPT: Considering the Implications of Generative Artificial Intelligence for Journalism and Media Education. *Journalism & Mass Communication Educator.* 2023;78(1):84–93. 10.1177/10776958221149577

[ref40] Peng Y Liu E Peng S : Using artificial intelligence technology to fight COVID-19: A review. *Artif. Intell. Rev.* 2022;55(6):4941–4977. 10.1007/s10462-021-10106-z 35002010 PMC8720541

[ref41] Perkins M : Academic Integrity considerations of AI Large Language Models in the post-pandemic era: ChatGPT and beyond. *J. Univ. Teach. Learn. Pract.* 2023a;20(2). 10.53761/1.20.02.07

[ref58] Perkins M : Generative AI policies for academic publishers. figshare.[dataset].2023b. 10.6084/m9.figshare.24124860.v1

[ref42] Perkins M Roe J : Decoding Academic Integrity Policies: A Corpus Linguistics Investigation of AI and Other Technological Threats. *High Educ. Pol.* 2023. 10.1057/s41307-023-00323-2

[ref43] Perkins M Roe J Postma D : Detection of GPT-4 Generated Text in Higher Education: Combining Academic Judgement and Software to Identify Generative AI Tool Misuse. *Journal of Academic Ethics.* 2023. 10.1007/s10805-023-09492-6

[ref44] Pichai S : *An important next step on our AI journey.* Google;2023, February 6. Reference Source

[ref45] Pourhoseingholi MA Hatamnejad MR Solhpour A : Does chatGPT (or any other artificial intelligence language tool) deserve to be included in authorship list? *Gastroenterol. Hepatol. Bed Bench.* 2023;16(1):435–437. 10.22037/ghfbb.v16i1.2747 37070106 PMC10105502

[ref46] Rahimi F Talebi Bezmin Abadi A : ChatGPT and Publication Ethics. *Arch. Med. Res.* 2023;54(3):272–274. 10.1016/j.arcmed.2023.03.004 36990890

[ref47] Risko EF Gilbert SJ : Cognitive Offloading. *Trends Cogn. Sci.* 2016;20(9):676–688. 10.1016/j.tics.2016.07.002 27542527

[ref48] Roe J Perkins M : What are Automated Paraphrasing Tools and how do we address them? A review of a growing threat to academic integrity. *Int. J. Educ. Integr.* 2022;18(1):Article 1. 10.1007/s40979-022-00109-w

[ref49] Roe J Renandya W Jacobs G : A Review of AI-Powered Writing Tools and Their Implications for Academic Integrity in the Language Classroom. *J. Eng. Appl. Linguist.* 2023;2(1). 10.59588/2961-3094.1035

[ref50] Salvagno M Taccone FS Gerli AG : Can artificial intelligence help for scientific writing? *Crit. Care.* 2023;27(1):75. 10.1186/s13054-023-04380-2 36841840 PMC9960412

[ref63] Shaw D Satalkar P : Researchers’ interpretations of research integrity: A qualitative study. Accountability in Research 2018;25(2):79–93. 10.1080/08989621.2017.1413940 29291621

[ref51] Solomon DH Allen KD Katz P : Artificial Intelligence, Authorship, and Medical Publishing. *ACR Open Rheumatol.* 2023;5(6):288–289. 10.1002/acr2.11538 37036239 PMC10267801

[ref53] Tan P Chen X Zhang H : Artificial intelligence aids in development of nanomedicines for cancer management. *Semin. Cancer Biol.* 2023;89:61–75. 10.1016/j.semcancer.2023.01.005 36682438

[ref54] Tate T Doroudi S Ritchie D : Educational Research and AI-Generated Writing: Confronting the Coming Tsunami. 2023. 10.35542/osf.io/4mec3

[ref57] Weber-Wulff D Anohina-Naumeca A Bjelobaba S : Testing of detection tools for AI-generated text. *International Journal for Educational Integrity.* 2023;19: Article 1. 10.1007/s40979-023-00146-z

[ref55] Wikipedia: Rankings of academic publishers. *Wikipedia.* 2023. Reference Source

[ref56] Xiao P Chen Y Bao W : Waiting, Banning, and Embracing: An Empirical Analysis of Adapting Policies for Generative AI in Higher Education (SSRN Scholarly Paper 4458269). 2023. 10.2139/ssrn.4458269

