# Supplementary material for: Academic publisher guidelines on AI usage: A ChatGPT supported thematic analysis
Source: F1000Res. 2024 Jan 16;12:1398. Originally published 2023 Oct 23. [Version 2] doi: 10.12688/f1000research.142411.2 (PMC10844801; doi:10.12688/f1000research.142411.2)
Supplement: Peer review history

# Reviewer report: Reviewer response for version 2

**DOI:** 10.5256/f1000research.160745.r238282

**Published:** 2024-02-05

**Authors:** Salim Razi

**Affiliations:** 1 English Language Teaching, Canakkale Onseikz Mart University, Çanakkale, Turkey

I would like to thank the authors for carefully addressing my comments in the revised version of their article. The article reads quite well to me and I do not have any further comments. I am pretty sure the readers will find the article valuable.

Is the work clearly and accurately presented and does it cite the current literature?

Yes

If applicable, is the statistical analysis and its interpretation appropriate?

Yes

Are all the source data underlying the results available to ensure full reproducibility?

Yes

Is the study design appropriate and is the work technically sound?

Yes

Are the conclusions drawn adequately supported by the results?

Yes

Are sufficient details of methods and analysis provided to allow replication by others?

Yes

Reviewer Expertise:

Academic integrity policies, plagiarism prevention, text-matching tools

I confirm that I have read this submission and believe that I have an appropriate level of expertise to confirm that it is of an acceptable scientific standard.

# Reviewer report: Reviewer response for version 2

**DOI:** 10.5256/f1000research.160745.r238281

**Published:** 2024-02-05

**Authors:** Brenda M. Stoesz

**Affiliations:** 1 University of Manitoba, Winnipeg, Manitoba, Canada

The authors have addressed the concerns appropriately.

Is the work clearly and accurately presented and does it cite the current literature?

Yes

If applicable, is the statistical analysis and its interpretation appropriate?

Yes

Are all the source data underlying the results available to ensure full reproducibility?

Yes

Is the study design appropriate and is the work technically sound?

Yes

Are the conclusions drawn adequately supported by the results?

Yes

Are sufficient details of methods and analysis provided to allow replication by others?

Partly

Reviewer Expertise:

Academic integrity, research integrity, policy analysis

I confirm that I have read this submission and believe that I have an appropriate level of expertise to confirm that it is of an acceptable scientific standard.

# Reviewer report: Reviewer response for version 2

**DOI:** 10.5256/f1000research.160745.r238284

**Published:** 2024-02-05

**Authors:** Stephen Gow

**Affiliations:** 1 University of York, York, England, UK

I am satsifed the authors have responded to my comments and appreciate the time they have taken to respond to the review. Many thanks.

Is the work clearly and accurately presented and does it cite the current literature?

Yes

If applicable, is the statistical analysis and its interpretation appropriate?

Yes

Are all the source data underlying the results available to ensure full reproducibility?

Yes

Is the study design appropriate and is the work technically sound?

Yes

Are the conclusions drawn adequately supported by the results?

Yes

Are sufficient details of methods and analysis provided to allow replication by others?

Yes

Reviewer Expertise:

Academic Integrity, Assessment, Policy as they relate to generative AI.

I confirm that I have read this submission and believe that I have an appropriate level of expertise to confirm that it is of an acceptable scientific standard.

# Reviewer report: Reviewer response for version 1

**DOI:** 10.5256/f1000research.155964.r218377

**Published:** 2023-11-20

**Authors:** Salim Razi

**Affiliations:** 1 English Language Teaching, Canakkale Onseikz Mart University, Çanakkale, Turkey

The manuscript reports an inductive thematic analysis by investigating 29 publisher policies regarding AI-assisted authorship and academic work. It is well-written in an engaging and lively style on a hot topic to which they make significant contributions. The authors produced a number of positive and welcome outcomes including the literature review which offers a useful overview of current research and policy and the resulting bibliography which provides a very useful resource for further researchers, policy makers, and publishers. In general, I found the manuscript quite good; however, I would like to call the authors’ attention to a few minor issues that I list below.

 Page 3: The authors use both “academic integrity” and “research integrity” in the first paragraph of the introduction section. It would be nice to inform readers about the interaction of these two related terms. I see that the authors make use of these two terms quite often in the rest of the paper.

 Page 3: Considering the objectives and structure of the study, the authors indicate that they explored “the policies of 109 academic publishers who are key stakeholders in the production of scholarly work”. Did they include any policy that they were able to find in their analysis or did they have any inclusion or exclusion criteria for the inclusion of policies in their corpus? Although the authors provide more explanation under methods section, I wish to see inclusion and exclusion criteria clearly.

 Page 4: “‘brace for impact’ (Pichai, 2023, as cited in Elias, 2023)”. Page numbers need to be provided for direct quotations. In addition, the authors use single quotation marks both to present terminology and to show direct quotations which I found quite difficult to interpret.

 Page 5: “the use of Large Language Models (LLMs).” LLM was already abbreviated on Page 3.

 Page 5: “At the core of the issues of academic integrity and ethics.” To my understanding, academic integrity already covers ethics; so, do we need to mention it separately?

 Page 6: “it is crucial that journal publishers bolster plagiarism detection systems”. I would suggest the authors to avoid using “plagiarism detection systems” since it gives the impression that these systems can detect plagiarism automatically without a careful investigation of matches by an expert.

 Page 7: “In our methodological process, these analyses were conducted separately by the two researchers to [establish intercoder agreement].” I suggest adding the phrase in brackets.

 Page 7: “(Boyatzis, 1998, p1)” should be “(Boyatzis, 1998, p. 1)”.

 Page 8: “Elsevier will monitor…” missing full stop.

 Page 8: “the politics of detection”. I guess this is not “politics” but “policies”.

 Page 8: The authors are referring to the policies of MDPI under “manual analysis theme three”. Previously, the authors indicated that they did not include predatory publishers in the analysis. I am not labelling any publisher as possibly predatory here; however, I would like to call the authors’ attention to “Predatory Reports” available at https://predatoryreports.org/news/f/list-of-all-mdpi-predatory-publications .

 Page 10: “the advanced data analysis tool demonstrated significantly different results.” I would invite the authors to remove “significantly” from this expression as “significant” is a statistical term making a reference to “p” values in quantitative analysis.

 Page 11: “The final themes for the AI…” missing full stop.

 Page 14: “then used to further enrich and triangulate findings.” Since this makes a reference to qualitative studies, “findings” should be replaced with “results.”

 Page 15: “Dawson (2022) as cited in Sparrow, 2022” should be “Dawson, 2022 as cited in Sparrow, 2022”.

Is the work clearly and accurately presented and does it cite the current literature?

Yes

If applicable, is the statistical analysis and its interpretation appropriate?

Yes

Are all the source data underlying the results available to ensure full reproducibility?

Yes

Is the study design appropriate and is the work technically sound?

Yes

Are the conclusions drawn adequately supported by the results?

Yes

Are sufficient details of methods and analysis provided to allow replication by others?

Yes

Reviewer Expertise:

Academic integrity policies, plagiarism prevention, text-matching tools

I confirm that I have read this submission and believe that I have an appropriate level of expertise to confirm that it is of an acceptable scientific standard, however I have significant reservations, as outlined above.

# Response

**Published:** 2023-12-28

**Authors:** Mike Perkins

Thank you very much for your detailed review. Although we do not agree with every one of your points, we have clarified where this is the case in the points below.

1. This is definitely useful, and we have added a small section in the introduction referring to how we interpret the term research integrity.
2. We have adjusted this section to clarify-107 publishers was our total population (following the exclusion of known predatory publishers). Any publishers with policies mentioning GenAI were then analysed in more detail.  
3. No page numbers are available for this online source. Single quotation marks are used throughout as a stylistic choice consistent with British English.
4. Abbreviations have been checked and adjusted as needed. Policy quotes have been left unchanged.
5. Changed to focus on research integrity.
6. This statement has been removed as it was unclear-we are referring to GenAI detection which has been demonstrated to be inaccurate and have provided additional citations for this.
7. In typical qualitative research, this may be the goal, but here we are specifically focused on the differences between the multiple analyses carried out. We were not therefore seeking to establish inter-coder agreement (although this did occur).
8. Corrected
9. Corrected
10. Corrected!
11. We had discussions regarding the inclusion/exclusion of MDPI as a potential predatory publisher and made the decision not to include this, primarily based on the most updated version of Beall’s list.
12. The use of ‘significant’ is not a term that is restricted solely to statistical usage; however, we have replaced this with ‘major’ to avoid any confusion.
13. Corrected
14. Thank you, but we prefer the original wording-we are referring to findings in the sense of the interpreted results of multiple human and GenAI supported analyses. Any GenAI supported analysis requires human-input and interpretation, therefore we believe that findings is the more appropriate term here given that the raw ‘results’ may not be accurate
15. This reference has been replaced with an alternative primary source from Dawson.

# Reviewer report: Reviewer response for version 1

**DOI:** 10.5256/f1000research.155964.r218378

**Published:** 2023-11-15

**Authors:** Stephen Gow

**Affiliations:** 1 University of York, York, England, UK

Overall, I found this paper timely, innovative and thought-provoking. It makes a solid contribution to the use of generative AI in research and for publication, and is of use for policy makers, researchers and editors alike. In a suitably meta approach, the papers carries out both of the following: 

1. The identification and thematic analysis of 28 academic publishers’ policy response to generative AI
2. The hybrid human and generative AI analysis of these policies in order to demonstrate and evaluate the use of generative AI for policy/qualitative research.

The paper clearly states and meets 4 objectives it set’s out. My main take away is the potential use of generative AI to support research, as the authors say, with “human intervention, oversight and a robust triangulation process…” - I expect some of this message may be lost in the summarisation of this article in media - it is not the simple case of ChatGPT carrying out the research, it is carefully used as a tool but human intuition is needed. This actually reinforces the key theme identified in the thematic analysis of the policies - *human exclusive authorship* - at the current time. It is apparent at this stage that generative AI requires significant human input in order to avoid errors and fabrications. My second take away is that the there is a *fluid policy landscape* in which publishers are attempting to allow for the use of generative AI but this is highly complex due to the human exclusive authorship and the need for transparency to clearly demarcate where humans and AI contribute to the research.

 I would certainly recommend researchers, teachers and policy makers to read the paper, however I do have the following 10 areas of critique which I hope the authors can take on board:

1. **Progressive vs Conservative approach to AI: **The authors are clearly in favour of utilising generative AI for the purposes of research, this bias is strong in the paper - with them referring to the “knee jerk reaction” or reactionary nature of policies which do not embrace generative AI as wholeheartedly as they do. They do however acknowledge the *fluid policy landscape* as a key theme. In this the authors note the publishers' flexibility to adapt to the rapidly changing nature of the technology and its impact on academic publishing. While the paper clearly notes that publishers are not attempting to ban AI and do see the potential benefits, the policies are clearly not as progressive as the authors would like. The authors call for more nuanced policies based on AI’s capabilities and limitations, though this is easier said than done as much guidance at this stage is speculative and quickly outdated. This is where I would use the terms conservative and cautious rather than reactionary, they seem like more fair terms for the publishers. Publishers are considering not only authorship and ethical considerations but legal risks involved in publishing work which they may be culpable for in the future, which provides extra cause for caution. 
2. **AI as an entirely new problem for authorship/academic integrity: **Judging on the literature referred to in the paper, one may get the impression that questions of authorship and AI are completely new in the past two years. This is not the case as authorship and AI has been discussed, particularly in legal journals, for many years pre-dating ChatGPT’s release. In relation to point 1., this is why it is strange that publishers are suddenly aware of this issue as it has clearly been emerging for many years, for example GPT-3 was reasonably capable a full 2 years prior to Chat-GPT. Furthermore, the references do not fully place the research in the rich body of scholarship of the impact of technology on authorship and academic integrity, the arms race analogy of academic authorship dates back to until at least 2000, even then Betty Leask (2007) discusses why war metaphors may not be helpful with discussions of students plagiarism, I expect similar in this case.  
3. **Self-referential:** In relation to point 2, the authors refer to the own work a lot - I know from reading their previous work that they are aware of the literature around technology, authorship and academic integrity. I suspect a rush to publish the work in a fast moving field may be partly behind this, so hope the authors may further contextualise their work within the relevant scholarship, I have provided some suggestions below. 
4. **Transparency: **The research use of generative AI is clearly set out, however the use of generative AI for drafting is not. This is related to the key theme of *transparency in Ai utilisation* which they identify. As with any writing now, I am curious about whether something has been written by generative AI and especially so with this paper where is admitted it has been used, the demarcation is not clear - a little more explanation of this may help and help for developing a *disclosure mandate *as suggested. Even if AI has been used in the drafting, the authors have clearly used it in a responsible manner and it reads well - but, in regards to point 3, if writing has been drafted with AI and references added in after, this raises questions about which references the AI is drawing from, this also relates to the the ethical and copyright concerns of using generative AI at all.
5. **Impact of Generative AI on the research process: **The authors clearly state how generative AI has been used, it helped identify both obvious and subtle codes in the data and assisted with theme development. I am curious as to whether it has any advantages over other Computer Assisted Qualitative Data Analysis Software, such as Nvivo? I also question whether the researchers would have come up with radically different conclusions had they not used ChatGPT or used an alternative software. The authors did however compare different versions of ChatGPT, which was useful.
6. **Differentiation of AI/Machine learning and Generative AI:** It seems to be the generative element of AI that leads to the problems with it on a practical and theoretical level - e.g. can it be accountable. With non-generative AI/machine learning models - the function is more specific and issues of authorship less problematic, which raises the question about whether generative AI has actually hindered the adaptation of the world to AI and caused the knee jerk reaction. 
7. **Copyright issues of how generative AI is trained:** Though the authors discuss ethical and copyright issues, a large part of the debate is the copyright or the data used to train models, I don’t feel this is given enough attention given the authors are using the software.
8. **Minor error:** Paper states on p3. That there is an in-depth exploration of 109 academic publishers however - the in-depth analysis is only carried out on 28 policies, as far as I can tell.
9. **Cognitive offloading: **Why is Dawson’s work referenced with a secondary citation? It’s quite accessible. 
10. **Further research: **I’d love to see a similar approach for national and international level university policy - I’d be very interested in working on this!

Suggested papers and some I refer to in the review which may be of interest:

- Beer, D. (2019) - Should we use AI to make us quicker and more efficient researchers? *LSE Impact of Social Sciences Blog* https://blogs.lse.ac.uk/impactofsocialsciences/2019/10/30/should-we-use-ai-to-make-us-quicker-and-more-efficient-researchers/
- Cole, S., & Kiss, E. (2000). What can we do about student cheating. About Campus, 5(2), 5-12. - mentions the arms race in terms of technology and academic integrity.
- Dawson, P. (2020). Cognitive offloading and assessment. Re-imagining university assessment in a digital world, 37-48.
- Eaton, S. E. E. (2023). 6 Tenets of Postplagiarism: Writing in the Age of Artificial Intelligence [Infographic]. https://prism.ucalgary.ca/items/86a7af9b-fd25-4d35-b20e-cf225fa2d3f5 
- Gervais, D. J. (2019). The machine as author. Iowa L. Rev., 105, 2053. 
- Leask, B. (2006). Plagiarism, cultural diversity and metaphor—implications for academic staff development. Assessment & Evaluation in Higher Education, 31(2), 183-199.
- Rogerson AM, McCarthy G (2017) Using internet based paraphrasing tools: original work, patchwriting or facilitated plagiarism? International Journal for Educational Integrity 13:2
- Schafer, B., Komuves, D., Zatarain, J. M. N., & Diver, L. (2015). A fourth law of robotics? Copyright and the law and ethics of machine co-production. Artificial Intelligence and Law, 23, 217-240. https://link.springer.com/article/10.1007/s10506-015-9169-7

Is the work clearly and accurately presented and does it cite the current literature?

Yes

If applicable, is the statistical analysis and its interpretation appropriate?

Yes

Are all the source data underlying the results available to ensure full reproducibility?

Yes

Is the study design appropriate and is the work technically sound?

Yes

Are the conclusions drawn adequately supported by the results?

Yes

Are sufficient details of methods and analysis provided to allow replication by others?

Yes

Reviewer Expertise:

Academic Integrity, Assessment, Policy as they relate to generative AI.

I confirm that I have read this submission and believe that I have an appropriate level of expertise to confirm that it is of an acceptable scientific standard, however I have significant reservations, as outlined above.

## References

[1] : Should we use AI to make us quicker and more efficient researchers?. *LSE Impact of Social Sciences Blog* .2019; Reference source

[2] : What Can we do about Student Cheating. *About Campus: Enriching the Student Learning Experience* .2000;5(2) : 10.1177/108648220000500203 5-12 10.1177/108648220000500203

[3] : Integrated mRNA and small RNA sequencing reveals post-transcriptional regulation of the sesquiterpene pathway in Santalum album L. (Indian sandalwood). *3 Biotech* .2023;13(12) : 10.1007/s13205-023-03816-4 387 10.1007/s13205-023-03816-4 37942052 PMC10628100

[4] : 6 Tenets of Postplagiarism: Writing in the Age of Artificial Intelligence [Infographic].2023; Reference source

[5] : The machine as author. *Iowa L. Rev* .2019;105:2053-2106

[6] : Plagiarism, cultural diversity and metaphor—implications for academic staff development. *Assessment & Evaluation in Higher Education* .2006;31(2) : 10.1080/02602930500262486 183-199 10.1080/02602930500262486

[7] : Using Internet based paraphrasing tools: Original work, patchwriting or facilitated plagiarism?. *International Journal for Educational Integrity* .2017;13(1) : 10.1007/s40979-016-0013-y 10.1007/s40979-016-0013-y

[8] : A fourth law of robotics? Copyright and the law and ethics of machine co-production. *Artificial Intelligence and Law* .2015;23(3) : 10.1007/s10506-015-9169-7 217-240 10.1007/s10506-015-9169-7

# Response

**Published:** 2023-12-28

**Authors:** Mike Perkins

Thank you very much for your thought provoking review. Although we do not agree with every one of your points, we have clarified this below in the point-by-point response.

  

1. A very fair point-we are pushing what is quite a major change to traditional academic practices and have adjusted the language as suggested.  Reactionary is left in one case as it is used in the sense of making a policy decision that is not informed by research and a full understanding.
2. We have expanded the volume of references around the ‘arms race’ approach to integrity. We recognise the perspective offered by Betty Leask, but believe this is a relevant analogy-the addition of further references does demonstrate the somewhat common nature of this in the literature.
3. We have broadened the citation scope to include a wider range of scholarship, including some of the specific texts mentioned. Although the ‘machine as author’ argument is an interesting area to explore, we are focusing the work in this case towards the applicability of using these GenAI tools for supporting research efforts.
4. There is an AI usage disclaimer present which sets out how GenAI has been used in this study. Yes, it was used to generate draft text, but for the majority of cases, this was a starting point with rewritings and revisions occurring iteratively to get to the final version. We feel that the current disclosure statement goes beyond the minimum disclosure of the use of the tools to focus specifically on the tasks that were carried out. Going beyond this would, we believe, be excessive. To what extent Ai disclaimers will be necessary in the near future is also an interesting point for discussion-as these tools continue to gain prevalence and acceptance in society, will the need to mention them continue to be a requirement form publishers? As per the discussions on cognitive offloading, we don't mention that we used spell checkers or grammar checkers so where is the line? Again, the ethical and copyright concerns of using AI are a little beyond the scope of this paper.
5. While qualitative data analysis software (e.g. NVivo) is exceptionally helpful for research purposes, we would argue that it can be used in conjunction with GenAI tools. The primary reason for this is that GenAI tools enable a triangulation – they can produce a summary of data analysis and interpretation that can be checked against the researcher-led output derived using tools such as NVivo. Furthermore, although these tools have the potential for user confirmation bias (as ‘stochastic parrots’), there is an advantage to having a GenAI ‘research partner’ or collaborator with which ideas can be experimented. It is possible that using different GenAI tools could have led to different results; this is an area that requires further study in the future but we do not feel invalidates our approach and results.
6. Yes, this is certainly one reason! Others would be the lack of reproducibility of the results, and the issues with hallucination (even challenges with changing the original dataset)
7. Again, this is a much broader issue that is not really within the scope of our paper to address. We refer to the European Commission’s point that copyright implications are still currently unclear (EC, 2023) with GenAI tools such as ChatGPT and the training data used on the model. Thus, while we acknowledge this issue, we do not specifically address it within the scope of this paper. Rather, we have focused the discussion of copyright around authorial rights and obligations, not the ethics of the tools themselves. Simply using the software does not, in our opinion necessitate a discussion around the ethics of the creation of the tool. European Commission (2023) Intellectual Property in ChatGPT. Available at https://intellectual-property-helpdesk.ec.europa.eu/news-events/news/intellectual-property-chatgpt-2023-02-20_en.
8. This is clarified later on, but we have adjusted the text here to make the methodology clearer. We reviewed 107 policies, and carried out an in depth analysis on the 28 which made reference to GenAI tools.
9. This news article cites Dawson specifically discussing GenAI tools as a method of cognitive offloading. However, given that other reviewers have also commented on this, we have replaced this citation with Dawson’s original work on cognitive offloading for clarity and to avoid further confusion.
10. That would definitely be an interesting paper-lets discuss more!

# Reviewer report: Reviewer response for version 1

**DOI:** 10.5256/f1000research.155964.r218375

**Published:** 2023-11-13

**Authors:** Mary Davis

**Affiliations:** 1 Oxford Brookes Business School, Oxford, UK

A great deal of current research is focusing on the impacts of AI in academia. The authors make an original contribution by identifying a specific area of importance to researchers: current publisher guidance on use of AI. They focus on an analysis of 28 academic publishers’ guidelines on use of AI in author submissions to establish the main themes prevalent in this guidance. By using an AI-generated analysis to examine guidance on use of AI, as well as human analysis, the authors add an additional level of interest and originality to the paper. They provide a well-documented analysis and discussion leading to the emergence of the final themes in publisher guidance on AI: human-exclusive authorship, author accountability, disclosure and transparency, research integrity, fluid policy landscape and constraints and exclusions.

 I found this to be an interesting study which will be directly useful for researchers and publishers in terms of the emerging themes, but also to everyone interested in guidance on AI and indeed conducting research using AI. It was good to see the linked full data for the analysis with the researchers' notes.  I recommend the authors consider some minor revisions for this paper as follows:

**1. Abstract - Background line 4**

 ‘and prompting discussions’ – this seems ambiguous in the sentence. If it is the lack of policies which prompt discussions, I suggest revising as ‘which has prompted discussions among publishers…’

**2. Introduction paragraph 1**

 In your introduction to key terms, some more definitions would be useful; in particular, I recommend adding whether you are using the terms ‘GenAI tools’ and ‘AI tools’ interchangeably or for different meanings throughout the paper.

**3. Introduction paragraph 2 line 6**

 ‘This has sparked a technological ‘arms-race’ – in fact reports of a technological arm’s race have been going a very long time in regard to plagiarism and text-matching tools, so rather than ‘sparked’, it might be better to talk about something like an ‘escalation’ in the ongoing arms race. For background, you may like to look at the work of Betty Leask nearly 20 years ago who talked about these battles in: Plagiarism, cultural diversity and metaphor - implications for staff development *Assessment & Evaluation in Higher Education* Vol. 31, No. 2, April 2006, pp. 183–199.

**4. Literature review - AI’s role and ethical implications in academic research - Paragraph 2 line 8**

 ‘Pass GenAI texts on their own’ – I think you mean ‘pass off GenAI texts as their own’?

**5. Literature review - AI’s role and ethical implications in academic research – Paragraph 3**

 Consider avoiding repetition of ‘perpetuate’ and ‘perpetuating’ lines 2-3.

**6. Results section one – line 1**

 You mention the manual analysis was carried out using QDA Miner software, so actually the manual analysis was not fully manual. The user guide says  it can be used for ‘coding textual and graphical data, annotating, retrieving and reviewing coded data, documents and images’ https://provalisresearch.com/uploads/QDA-Miner-5-User-Guide-V1.2.pdf

  It would be helpful to the reader for you to clarify further how the software was employed in this study.

**7. Results section one - Manual analysis subsections**

 You don’t provide any tables for your manual analysis calculations in this part, you just incorporate them in table 4 to compare with the AI analysis. It would be helpful to explain or at least signpost to the reader that the data from the manual analysis can be seen in table 4.

**8. Table 3**

 I am wondering why the line ‘limitations and prohibitions’ is repeated across 2 lines at the bottom of the table?

**9. Limitations and implications – paragraph 2 line 11**

 I suggest citing Dawson directly rather than involving Sparrow’s article in the Guardian, unless you want to highlight newspaper discussion.

**10. References**

 Bealls list.net, no date – I would suggest Beall’s List, 2021 – the date given on the website for the most recent update.

Is the work clearly and accurately presented and does it cite the current literature?

Yes

If applicable, is the statistical analysis and its interpretation appropriate?

Yes

Are all the source data underlying the results available to ensure full reproducibility?

Yes

Is the study design appropriate and is the work technically sound?

Yes

Are the conclusions drawn adequately supported by the results?

Yes

Are sufficient details of methods and analysis provided to allow replication by others?

Partly

Reviewer Expertise:

Academic integrity, plagiarism, inclusion, Artificial Intelligence

I confirm that I have read this submission and believe that I have an appropriate level of expertise to confirm that it is of an acceptable scientific standard, however I have significant reservations, as outlined above.

## References

[1] : Plagiarism, cultural diversity and metaphor—implications for academic staff development. *Assessment & Evaluation in Higher Education* .2006;31(2) : 10.1080/02602930500262486 183-199 10.1080/02602930500262486

# Response

**Published:** 2023-12-28

**Authors:** Mike Perkins

Thank you very much for your review. Please see below for a point-by-point response.

1. Adjusted.
2.  AI tools are different to GenAI tools and are used as separate terms in the paper. Further definitions are provided in the literature review, but we choose to not dwell so much on definitions, given the specific objectives of this paper.
3. We have replaced 'sparked' with 'escalated' to more accurately represent the continuous development of the technological arms race in plagiarism detection and added additional citations for this statement.
4. Adjusted.
5. Replaced perpetuating with escalating.
6. This is correct, we have updated it to make it clear that this is an analysis assisted through QDA Miner software at the beginning of the paper, and when we discuss ‘manual’ we are referring to with use of non-GenAI software.
7. We have added signposting language to show where the results are for this section in Table 4.
8. This repeated row is deleted.
9. This news article cites Dawson specifically discussing GenAI tools as a method of cognitive offloading. However, given that other reviewers have also commented on this, we have replaced this citation with Dawson’s original work on cognitive offloading for clarity and to avoid further confusion.
10. Updated in the text and citation.

# Reviewer report: Reviewer response for version 1

**DOI:** 10.5256/f1000research.155964.r218374

**Published:** 2023-11-01

**Authors:** Brenda M. Stoesz

**Affiliations:** 1 University of Manitoba, Winnipeg, Manitoba, Canada

With the emergence of publicly accessible generative artificial intelligence (genAI) tools over the past year, concerns about authorship in research and research articles has emerged. The authors provide a brief background on this technological advancement and question how the policies of publishers have begun to address concerns about genAI tools being listed as an author or otherwise used to support the development of research articles. The authors used two methods to examine 28 policies - human coding and use of genAI - and found similarities and differences in extracted themes using the two methods. Although genAI assisted analysis was useful in some ways, the authors concluded that human input in data analysis and interpretation are important. The authors discuss the ethical and practical implications of their findings as they relate to non-native English speakers and writers, students, and the academic community as a whole.

 Here are some minor points to consider when revising this manuscript:

1. Some reorganization of the methods section where thematic analysis is described is warranted as there appears to be some redundancy. Please revise, although this is a minor point.
2. The last sentence of results section 1 indicated that there were no differences between different categories of publishers during manual analysis. Please add details about these categories in the methods section - these details may be present in the methods section but perhaps they are not as salient as they should be.
3. I wonder if the authors considered how prolific use of genAI for conducting and writing up research might impact the interpretations made by systematic review articles and/or meta-analyses.
4. There are some punctuation errors and omissions in a few places, and some odd text (i.e., #here) in a few places that need correction.

Overall, this is an interesting study and readers, especially editors, researchers, and other members of the academic community involved in internal policy making will find this study useful.

Is the work clearly and accurately presented and does it cite the current literature?

Yes

If applicable, is the statistical analysis and its interpretation appropriate?

Yes

Are all the source data underlying the results available to ensure full reproducibility?

Yes

Is the study design appropriate and is the work technically sound?

Yes

Are the conclusions drawn adequately supported by the results?

Yes

Are sufficient details of methods and analysis provided to allow replication by others?

Partly

Reviewer Expertise:

Academic integrity, research integrity, policy analysis

I confirm that I have read this submission and believe that I have an appropriate level of expertise to confirm that it is of an acceptable scientific standard, however I have significant reservations, as outlined above.

# Response

**Published:** 2023-12-28

**Authors:** Mike Perkins

Thank you very much for your comments and review. Please see below for a point-by-point response.

1. This has been reviewed and edited for clarity.
2. We have added clarification on the categories of publishers considered to the methods section. All publisher categories are shown in the additional data
3. This was not something we had considered. Assuming that GenAI supported research is done with integrity and transparency, there should be no implications for any systematic reviews-the problem would come if there was no oversight and AI hallucinations were present. However, as the methodology we describe is supported by GenAI tools, rather than carried out by them, we do not believe this to be an area of concern.
4. The paper has been reviewed again for punctuation errors. The # marks are direct quotes from the inputs used in the GenAI tools-marks like this can be used to organise complex inputs to GenAI tools, (for example, separating sample text from instructions on what to do with it) as these tools generally do not process line breaks.
